# EGFR A859S alteration may predict a better response to third-generation EGFR-TKI treatment in advanced NSCLC

**DOI:** 10.1016/j.gendis.2025.101757

**Published:** 2025-07-02

**Authors:** Weiping Hong, Wenfan Fu, Ya Ma, Chenxuan Wang, Yang Xu, Jiani Yin, Jiaohui Pang, Qiuxiang Ou, Hua Bao, Jincui Gu, Baoxiu Li

**Affiliations:** Department of Oncology, Guangdong Sanjiu Brain Hospital, Guangzhou, Guangdong 510510, China; Department of Chest Surgery, Affiliated Cancer Hospital & Institute of Guangzhou Medical University, Guangzhou, Guangdong 510095, China; Geneseeq Research Institute, Nanjing Geneseeq Technology Inc., Nanjing, Jiangsu 210032, China; Pulmonary and Critical Care Medicine, The First Affiliated Hospital of Sun Yat-sen University, Guangzhou, Guangdong 510080, China; Department of Oncology, Guangzhou First People’s Hospital, South China University of Technology, Guangzhou, Guangdong 510180, China

The epidermal growth factor receptor (*EGFR*) A859S alteration is a rare variant in non-small cell lung cancer (NSCLC) that occurs in only ∼0.01% of cases^1^ and has been reported in few studies.^2^^,^^3^ To better understand its clinical relevance, we retrospectively analyzed 66,946 NSCLC patients from our next-generation sequencing (NGS) database, identifying 36,181 with *EGFR* variants and 18 (0.02%) with the A859S alteration. Among these, 12 samples were collected at baseline, 3 at disease progression after first-line EGFR-TKI treatment, and 3 with unknown disease status. We aimed to characterize the mutational landscape of A859S and assess survival outcomes in advanced NSCLC patients treated with first-line EGFR-tyrosine kinase inhibitors (TKIs).

The median age of the A859S cohort was 66 years (range: 55–83), and 44.4% of them were male. Adenocarcinoma was the most common histology (10/18), followed by squamous carcinoma (2/18); no adenosquamous carcinoma was observed (Table S1). NGS revealed 13 EGFR mutation sites: 11 missense and 2 exon 19 deletions (19del) (Fig. 1A). L858R was the most frequent concurrent mutation (72%), followed by 19del (17%) and T790M (11%) (Fig. 1B). One of the two T790M-positive patients had prior first-generation TKI treatment. The A859S variant allele frequency (VAF) ranged from 1.93% to 61.21%, suggesting possible subclonality. *EGFR* copy number variation (CNV) was seen in three patients. Other frequent mutations included *TP53* (33%) and several others (11% each), with CNVs in *CDK4* (28%), *MYC*, *CCND1*, and *MDM2* (11% each). C > A mutations predominated, followed by T > G and C > T, indicating a transversion–dominant profile (Fig. 1C). Among the 12 baseline samples, 8 had concurrent L858R; the rest included *EGFR* 19del, *KRAS* G12V, *TP53* frameshift, or no other known driver. It may be worth noting that the patients had a squamous histological type. To our knowledge, this is the first report of single *EGFR* A859S mutations in lung cancer.^2^ While the presence of A859S in a squamous NSCLC sample without other driver mutations raises interest, its isolated oncogenic potential remains speculative and requires further functional validation.

**Figure 1:**
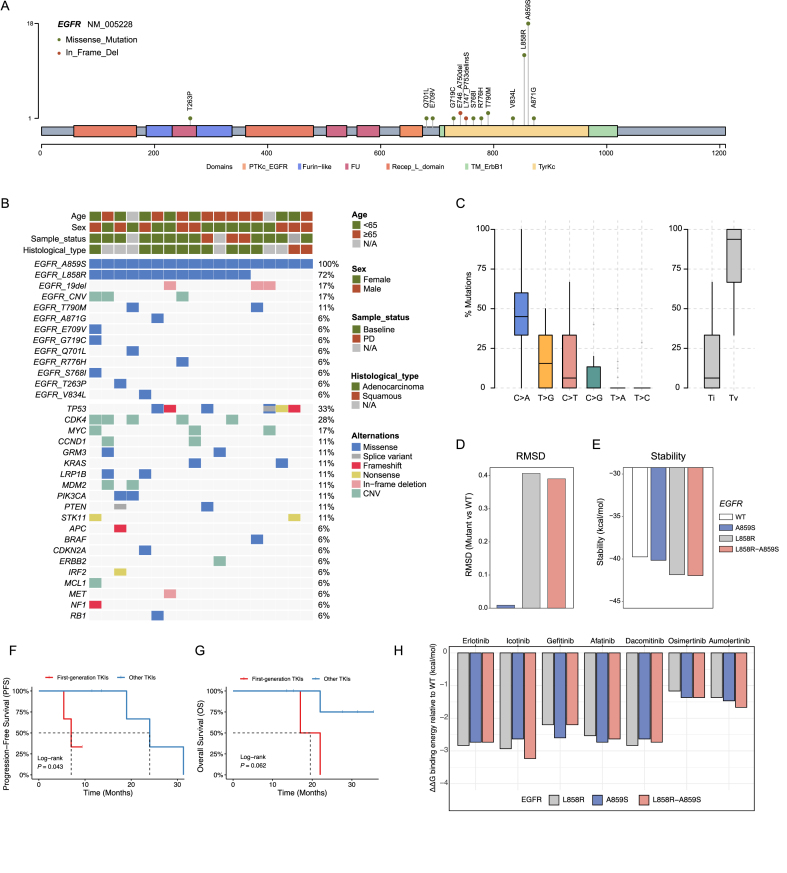
Overview of the characteristics of the *EGFR* A859S population. (**A**) Lollipop diagram displaying identified *EGFR* mutation sites. (**B**) Oncoplot showing the clinical and genomic profiles of the studied cohort (*n* = 18). Variations were divided into *EGFR* alterations and others, arranged according to mutation frequencies in the population. (**C**) Frequencies of point mutations, transitions, and transversions. (**D, E**) Protein structural simulations of *EGFR* A859S, L858R, and L858R-A859S. The template EGFR kinase domain in its active state (2GS2) was obtained from the Protein Data Bank, and YASARA was used to approximate structural variation and stability (kcal/mol). The root mean square deviation (RMSD) relative to that of wild-type (WT) was calculated for comparison of structural similarities. (**F, G**) Kaplan–Meier curves of the progression-free survival (PFS) and overall survival (OS) of the advanced NSCLC population with *EGFR* A859S alterations stratified by TKI treatment. (**H**) Binding affinities of EGFR tyrosine kinase inhibitors (TKIs) for L858R-, A859S-, and L858R-A859S-mutated *EGFR* relative to WT EGFR, as simulated using Autodock Vina. A more negative value indicates a higher binding affinity.

We assessed the oncogenic potential of the single A859S mutation using *in silico* modeling. Using the *EGFR* kinase domain structure from the Protein Data Bank and YASARA for simulation, we evaluated structural variation and stability. The L858R-A859S double mutant had an RMSD of 0.4071 relative to wild-type (WT) *EGFR* and closely resembled the L858R mutant, while the single A859S mutant was more similar to the WT (Fig. 1D). This may suggest that the replacement of alanine (non-polar, small) with serine (polar, hydroxyl-containing) in A859S introduces minimal steric hindrance, especially compared to the bulkier arginine of L858R. As a result, this subtle substitution does not deform the ATP-binding cleft, unlike L858R or 19del, which alter the hinge region and αC-helix orientation, facilitating increased affinity for ATP and TKIs. Regarding stability, prior studies have indicated that the L858R mutation stabilizes the αC-helix of the *EGFR* kinase domain, trapping the protein in an active conformation and enhancing oncogenicity.^4^ We found that the predicted stability of A859S-mutated *EGFR* (−40.13 kcal/mol) was closer to that of the WT *EGFR* (−39.76 kcal/mol), whereas L858R-A859S-mutated *EGFR* (−41.93 kcal/mol) exhibited stability comparable to that of L858R-mutated *EGFR* (−41.82 kcal/mol) (Fig. 1E). Both the L858R-A859S- and L858R-mutated forms were more stable than the WT (Fig. 1E), aligning with previous findings. It is likely that A859S does not occupy a position that influences ATP anchoring or the hydrophobic gatekeeper region like T790M, so its presence does not disrupt the ATP-competitive nature of most TKIs. Overall, A859S alone appears to be less oncogenic than L858R or L858R-A859S, though further experimental validation is needed.

Due to the limited survival data in our internal A859S cohort, we supplemented our analysis by including NSCLC patients from previous studies who had identified *EGFR* A859S mutations with available clinical information.^2^^,^^3^ This resulted in a cohort of eight patients for progression-free survival (PFS) analysis and eight patients for overall survival (OS) analysis (Table S2), and the objective response rate (ORR) was 75% (6/8). The median PFS (mPFS) was 21.5 months (7.0 months for first-generation TKIs and 24.0 months for later-generation TKIs), and the mOS was 22.0 months (Fig. 1F, G). In the PFS analysis cohort, four patients (50%) received 3rd-generation EGFR-TKIs, three received 1st-generation TKIs, and one received a 2nd-generation TKI (Table S2). Compared to published mPFS data for first-line EGFR-TKI treatments in advanced NSCLC patients,^5^ the mPFS of 21.5 months observed in the A859S cohort tended to be markedly longer. This trend was particularly notable in patients treated with 3rd-generation TKIs (Fig. 1F). To further investigate the impact of the A859S alteration on the efficacy of EGFR-TKIs, we performed structural analyses using AutoDock Vina to evaluate TKI binding to A859S-mutated and L858R-A859S-mutated *EGFR*. Docking simulations revealed higher binding affinities between 3rd-generation TKIs and *EGFR* with A859S alterations (Fig. 1H). Notably, aumolertinib showed greater binding affinity for A859S-mutated and L858R-A859S-mutated *EGFR* than for L858R-mutated *EGFR*, aligning with the prolonged PFS observed in the A859S cohort treated with this TKI.

This study has several limitations. First, its retrospective nature resulted in incomplete clinical information. Future studies with additional cases are needed to provide stronger evidence. Second, the structural predictions derived from *in silico* analyses may vary depending on the simulation methods used, potentially leading to conflicting results. Advancements in simulation methodologies, coupled with validation through *in vitro* experiments, are expected to enhance the reliability of these findings. Third, while our *in silico* analysis provides preliminary structural insights, limitations inherent to simulation-based methods must be acknowledged. Protein structures *in vivo* exhibit dynamic conformational changes that may not be fully captured in rigid docking models. Further validation through kinase activity assays, cellular proliferation models, and co–crystal structures is necessary to confirm the predicted affinities and stability changes. Fourth, we acknowledge that the absence of performance status (PS) data, limited PD-L1 expression reporting, and incomplete treatment histories for some patients introduce potential confounding factors. These gaps, combined with variable metastatic burdens and site involvement may influence the observed survival outcomes. Lastly, the small sample size significantly limits the statistical power and generalizability of our findings. This is especially important when interpreting survival data, which should be considered descriptive and hypothesis-generating rather than definitive. The potential for type I errors is elevated in this context, and the findings should be interpreted with caution. Similar interpretive challenges are well-documented in studies examining other rare *EGFR* mutations, such as L861Q and G719X. These variants often occur in compound form and have variable sensitivity to EGFR-TKIs, with outcomes depending on the mutation context, TKI generation, and co-mutation landscape.

In conclusion, this study characterized the clinical and genomic landscape of NSCLC patients harboring *EGFR* A859S mutations. Although trends suggest potential responsiveness to EGFR-TKI therapies, particularly third-generation agents, these results are preliminary and require validation in prospective, larger-scale studies. The structural simulations provide mechanistic hypotheses that should be confirmed through experimental approaches. Additionally, due to the retrospective design, limited cohort size, and incomplete clinical data, these observations should be interpreted with caution. The survival data presented are descriptive and exploratory and may be subject to confounding and type I error. Our findings are hypothesis-generating and underscore the need for larger-scale, multicenter studies and prospective clinical trials to further investigate the predictive significance and therapeutic responsiveness of rare *EGFR* mutations, including A859S.

## CRediT authorship contribution statement

**Weiping Hong:** Writing – review & editing, Writing – original draft, Visualization, Formal analysis, Data curation, Conceptualization. **Wenfan Fu:** Writing – review & editing, Writing – original draft, Formal analysis. **Ya Ma:** Writing – review & editing, Writing – original draft, Formal analysis. **Chenxuan Wang:** Writing – review & editing, Writing – original draft, Formal analysis. **Yang Xu:** Writing – review & editing, Writing – original draft, Formal analysis. **Jiani Yin:** Writing – review & editing, Writing – original draft, Formal analysis. **Jiaohui Pang:** Writing – review & editing, Writing – original draft, Formal analysis. **Qiuxiang Ou:** Writing – review & editing, Writing – original draft, Formal analysis. **Hua Bao:** Writing – review & editing, Writing – original draft, Formal analysis. **Jincui Gu:** Writing – review & editing, Writing – original draft, Supervision, Project administration, Conceptualization. **Baoxiu Li:** Writing – review & editing, Writing – original draft, Supervision, Project administration, Methodology, Investigation, Conceptualization.

## Ethics declaration

This study was approved by the Medical Ethics Committee of Nanjing Geneseeq Medical Laboratory (Ethics No. NSJB-MEC-2024-15). All specimens were collected with the informed consent of the patients.

## Data availability

The data generated or analyzed during this study are not publicly available due to ethical reasons but are available from the corresponding author upon reasonable request.

## Funding

This work was supported by the Science and Technology Program of Guangzhou, China (No. 202201010951) and the 2021 Science and Technology Foundation of Health Commission of Guangdong Province, China (No. gzwkj2021-055).

## Conflict of interests

Ya Ma, Yang Xu, Jiani Yin, Jiaohui Pang, and Hua Bao are employees of Nanjing Geneseeq Technology Inc. The remaining authors declare no conflicts of interest.
