# Supplementary material for: EGFR A859S alteration may predict a better response to third-generation EGFR-TKI treatment in advanced NSCLC
Source: Genes Dis. 2025 Jul 2;13(1):101757. doi: 10.1016/j.gendis.2025.101757 (PMC12451320; doi:10.1016/j.gendis.2025.101757)
Supplement: Multimedia component 1 [file mmc1.docx]

**Supplementary methods:**

**Study design**

We retrospectively analyzed next-generation sequencing (NGS) database of 66,946 non-small cell lung cancer (NSCLC) patients from Guangdong Sanjiu Brain Hospital, Affiliated Cancer Hospital & Institute of Guangzhou Medical University, The First Affiliated Hospital of Sun Yat-sen University, and Guangzhou First People's Hospital. A total of 36,181 patients harbored *EGFR* variations, and 18 (0.02%) of the patients had *EGFR* A859S alterations. Specifically, 12 samples were collected at baseline, 3 were collected at disease progression following first-line EGFR-TKI treatment, and the remaining 3 samples had no identifiable disease state. Clinical and genetic data of these 18 patients were proceeded to further analysis.

**DNA extraction and library preparation**

Genomic DNA was extracted from formalin-fixed and paraffin-embedded (FFPE) specimens using the QIAamp DNA FFPE Tissue Kit (Qiagen) according to the manufacturer’s protocol^1^. Blood samples were processed within 24 hours of collection by centrifugation to separate plasma and sediment, and plasma DNA was then fragmented into 300~350 bp using Covaris M220 instrument (Covaris). The quantity and quality of the extracted DNA were evaluated with a Qubit 3.0 fluorometer and Nanodrop 2000 (Thermo Fisher Scientific), respectively. Sequencing libraries were prepared using the KAPA Hyper Prep Kit (KAPA Biosystems) according to the manufacturer’s suggestions for different sample types. In brief, 1-2 μg of fragmented DNA underwent end-repairing, A-tailing, ligation with indexed adapters, and size selection using Agencourt AMPure XP beads (Beckman Coulter). Customized xGen lockdown probe panel (Integrated DNA Technologies) was used for targeted enrichment of predefined genes. The hybridization reaction was carried out with NimbleGen SeqCap EZ Hybridization and Wash Kit (Roche). Dynabeads M-270 (Life Technologies) was used to capture probe-bind fragments, followed by library amplification with Illumina p5 in KAPA HiFi HotStart ReadyMix (KAPA Biosystems), and purification by Agencourt AMPure XP beads. Library size distribution was measured by Agilent Technologies 2100 Bioanalyzer (Agilent Technologies). Enriched libraries were sequenced on Novaseq 6000 NGS platforms (Illumina).

**Sequencing data processing and bioinformatics analysis**

Trimmomatic was used for FASTQ file quality control (QC). Leading/trailing low quality (quality reading below 15) or N bases were removed. Reads from each sample were mapped to the reference sequence hg19 (Human Genome version 19) using Burrows-Wheeler Aligner (BWA-mem, v0.7.12). Local realignment around indels and base quality score recalibration were applied with the Genome Analysis Toolkit (GATK 3.4.0). GATK3.4.0 was applied to detect germline mutations from blood control samples. VarScan2 was employed to detect somatic mutations (somatic *P*-value = 0.1, minimum quality score = 15 and otherwise default parameters). Somatic variants analysis using Automated Triple Groom Sequencing (ATG-Seq) technology. Annotation was performed using ANNOVAR using the hg19 reference genome and 2014 versions of standard databases and functional prediction programs. A minimum VAF cutoff of 1% with a minimum of 100× depth and at least 5 supporting reads were used to call mutations, which is consistent with clinical-grade NGS pipelines.

***in silico* structure simulations**

To assess the oncogenic potential of the single *EGFR* A859S mutation, we performed in silico structural simulations. The active-state EGFR kinase domain (PDB ID: 2GS2) was retrieved from the Protein Data Bank and used as a structural template. All molecular modeling and simulations were conducted using YASARA, which enabled the evaluation of structural variations and stability changes upon mutation. The EGFR structures, including wild-type (WT), L858R, A859S, and L858R-A859S mutants, were subjected to energy minimization and refinement in YASARA to obtain optimized conformations. Structural deviations between the mutants and WT EGFR were quantified using root mean square deviation (RMSD) calculations. Stability assessments were performed by computing the total free energy of each structure, with lower energy values indicating greater stability. Drug-target binding affinities were analyzed using ConPLex^2^.

**Table S1** Patient characteristics.

| **PID** | **Age** | **Sex** | **Histological type** | **Sample type** | **Sample status** | **Firstline Treatment** |
| --- | --- | --- | --- | --- | --- | --- |
| P01 | 64 | Male | Adenocarcinoma | Tissue | Baseline | Immunochemotherapy |
| P02 | 63 | Male | Adenocarcinoma | Tissue | Baseline | Chemotherapy |
| P03 | 56 | Male | *N/A* | Tissue | Baseline | Surgery |
| P04 | *N/A* | Female | *N/A* | Liquid | *N/A* | *N/A* |
| P05 | 59 | Male | Squamous | Tissue | *N/A* | Brachytherapy & radiochemotherapy |
| P06 | 71 | Male | Squamous | Tissue | Baseline | *N/A* |
| P07 | 64 | Male | *N/A* | Tissue | Baseline | *N/A* |
| P08 | 82 | Female | Adenocarcinoma | Liquid | PD | Icotinib |
| P09 | 83 | Female | Adenocarcinoma | Liquid | Baseline | Aumolertinib |
| P10 | 66 | Female | Adenocarcinoma | Liquid | Baseline | Aumolertinib |
| P11 | 71 | Female | *N/A* | Tissue | *N/A* | *N/A* |
| P12 | 70 | Female | Adenocarcinoma | Tissue | PD | Dacomitinib |
| P13 | 50 | Female | Adenocarcinoma | Tissue | Baseline | Aumolertinib |
| P14 | 65 | Female | Adenocarcinoma | Tissue | PD | *N/A* |
| P15 | 67 | Male | Adenocarcinoma | Tissue | Baseline | Aumolertinib |
| P16 | 66 | Female | *N/A* | Tissue | Baseline | *N/A* |
| P17 | *N/A* | Female | *N/A* | Tissue | Baseline | *N/A* |
| P18 | 55 | Male | Adenocarcinoma | Tissue | Baseline | Surgery |

**Table S2** Patients for PFS and OS analysis.

| **Patient** | **First EGFR**  **TKI** | | **TKI generation** | | **Brain metastasis** | | **Other metastasis** | | **Treatment**  **history** | | **Co-mutated genes** | | **PD-L1**  **(22C3)** | | **PFS**  **status** | | **PFS**  **(month)** | | **OS**  **status** | | **OS**  **(month)** | | **For PFS analysis** | | **For OS analysis** | |  |
| --- | --- | --- | --- | --- | --- | --- | --- | --- | --- | --- | --- | --- | --- | --- | --- | --- | --- | --- | --- | --- | --- | --- | --- | --- | --- | --- | --- |
| Internal (P13) | Aumolertinib | | 3rd | | No | | No | | Aumolertinib for ~13 months (last follow-up: no PD) | | MET-exon19-del; TP53-frameshift | | <1% | | 0 | | 13.6 | | 0 | | 15.2 | | Yes | | Yes | |  |
| Internal (P15) | Aumolertinib | | 3rd | | No | | No | | Surgery+  Aumolertinib for ~11 months (last follow-up: no PD) | | CDK4-amp | | NA | | 0 | | 11.5 | | 0 | | 13.6 | | Yes | | Yes | |  |
| Internal (P09) | Aumolertinib | | 3rd | | No | | No | | Aumolertinib (for ~31 months; stopped Aumolertinib upon PD) | | BRAF-V600E; MYC-amp | | NA | | 1 | | 31.3 | | 0 | | 31.5 | | Yes | | Yes | |  |
| Internal (P10) | Aumolertinib | | 3rd | | No | | No | | Aumolertinib (for ~24 months, then stopped upon PD); then started dacomtinib | | RB1-L660V; RB1-Q395E; TP53-Y220D | | <1% | | 1 | | 24 | | 0 | | 27.6 | | Yes | | Yes | |  |
| Internal (P12) | dacomitinib | | 2nd | | No | | No | | Dacomitinib for 19 months (PD);  then Alflutinib for 15 months (PD) | | None | | NA | | 1 | | 19 | | 0 | | 35.5 | | Yes | | Yes | |  |
| Internal (P07) | Unspecified EGFR TKI | | N/A | | No | | No | | Unspecified  EGFR TKI | | KRAS-G12V; TP53-stop_gained | | NA | | 1 | | N/A | | 1 | | 22 | | No | | Yes | |  |
| External (Li, 2019^3^) | erlotinib | | 1st | | NA | | NA | | erlotinib | | TP53-missense | | NA | | 0 | | 9.4 | | N/A | | N/A | | Yes | | No | |  |
| External (Yang, 2020^4^) | icotinib | | 1st | | Yes | | No | | Icotinib (7 months; PD); then Osimertinib (80 mg per day for 8 months; PD);  then afatinib (40 mg per day for 2 months) | | None | | NA | | 1 | | 7 | | 1 | | 17 | | Yes | | Yes | |  |
| External (Abdulla, 2019^5^) | | erlotinib | | 1st | | Yes | | lymph node, thoracic vertebrae | | cisplatin/  pemetrexed; then erlotinib (150-300 mg/d) for 22 months | | None | | NA | | 1 | | 5.4 | | 1 | | 22 | | Yes | | Yes | |

**Reference**

1. Yang Z, Yang N, Ou Q, et al. Investigating Novel Resistance Mechanisms to Third-Generation EGFR Tyrosine Kinase Inhibitor Osimertinib in Non-Small Cell Lung Cancer Patients. Clin Cancer Res. 2018;24(13):3097-3107.

2. Singh R, Sledzieski S, Bryson B, Cowen L, Berger B. Contrastive learning in protein language space predicts interactions between drugs and protein targets. Proc Natl Acad Sci U S A. 2023;120(24):e2220778120.

3. Li M, Zhou CZ, Yang JJ, et al. The in cis compound EGFR mutations in Chinese advanced non-small cell lung cancer patients. *Cancer Biol Ther*. 2019;20(8):1097-1104. doi:10.1080/15384047.2019.1595280

4. Yang Y, Zhang X, Wang R, et al. Osimertinib Resistance With a Novel EGFR L858R/A859S/Y891D Triple Mutation in a Patient With Non-Small Cell Lung Cancer: A Case Report. *Front Oncol*. 2020;10:542277. doi:10.3389/fonc.2020.542277

5. Abdulla DSY, Scheffler M, Brandes V, et al. Monitoring Treatment Response to Erlotinib in EGFR-mutated Non-small-cell Lung Cancer Brain Metastases Using Serial O-(2-[(18)F]fluoroethyl)-L-tyrosine PET. *Clin Lung Cancer*. Mar 2019;20(2):e148-e151. doi:10.1016/j.cllc.2018.10.011
